# Advances in understanding nociception and neuropathic pain

**DOI:** 10.1007/s00415-017-8641-6

**Published:** 2017-10-14

**Authors:** Ewan St. John Smith

**Affiliations:** Department of Pharmacology, University of Cambridge, Tennis Court Road, Cambridge, CB2 1PD UK

**Keywords:** Chemogenetics, Neurocircuitry, Neuropathic pain, Nociceptor, Optogenetics, Voltage gated sodium channel (NaV)

## Abstract

Pain results from the activation of a subset of sensory neurones termed nociceptors and has evolved as a “detect and protect” mechanism. However, lesion or disease in the sensory system can result in neuropathic pain, which serves no protective function. Understanding how the sensory nervous system works and what changes occur in neuropathic pain are vital in identifying new therapeutic targets and developing novel analgesics. In recent years, technologies such as optogenetics and RNA-sequencing have been developed, which alongside the more traditional use of animal neuropathic pain models and insights from genetic variations in humans have enabled significant advances to be made in the mechanistic understanding of neuropathic pain.

## Introduction

Nociception is the neural process of encoding noxious stimuli, whereas pain is defined as an unpleasant sensory and emotional experience associated with actual or potential tissue damage, or described in terms of such damage [1]. Nociception has been described in a variety of organisms, from the nematode worm *Caenorhabditis elegans* through to humans, but the case for pain is less clear. Although humans and likely all mammals experience negative emotion, this is considered unlikely in *C. elegans*, but the case for certain organisms, especially fish, is more contentious [2–4]. Numerous reviews have been written about different aspects of pain, from its molecular basis [5–10] and genetic mechanisms [11–13] to its pharmacological treatment [14–16]. The purpose of this review is to discuss how recent insights into pain mechanisms from pre-clinical research may lead to breakthroughs in our understanding, and hopefully treatment, of chronic pain.

Chronic pain is usually defined as regularly occurring pain over a period of several months and it has a prevalence of ~11–19% of the adult population [17–19]. Broadly speaking, chronic pain can be split into two categories, inflammatory pain and neuropathic pain. Neuropathic pain is pain caused by a lesion or disease of the somatosensory nervous system and a systematic review of epidemiological studies estimates the prevalence of neuropathic pain to be 6.9–10% [20]. The need for novel therapies to treat neuropathic pain is demonstrated by the analysis of analgesia success. A 2006 report on chronic pain in Europe identified that 64% of those taking prescription medicine found that their pain medication was at times inadequate, and of the 48% of chronic pain sufferers not taking pain medication, 14% had stopped due to side effects [18]. To develop new treatments for neuropathic pain, it is important to first understand the circuitry of pain: how is pain triggered and how is that information transmitted to the central nervous system? To do this, it is necessary to understand how nociceptors function.

## Nociceptors: transducers of pain

The human body is equipped with different types of sensory neurones and nociceptors are the subset that function as the primary unit of pain, being equipped with receptors and ion channels that enable the detection of stimuli that have potential to cause damage. When a noxious stimulus activates an ion channel on a nociceptor, for example proton activation of acid-sensing ion channels (ASIC), cation influx depolarises the nociceptor producing a receptor potential. If the receptor potential is of sufficient magnitude to reach the activation threshold for voltage-gated Na^+^ channels (NaV), it will trigger action potential generation and transmission of a pain signal to the spinal cord [2, 5, 21]. In recent years, many new techniques have been developed in pre-clinical research that have accelerated our progress in understanding how nociceptors work and provide tantalising glimpses at their clinical utility. Indeed, such work is essential for both identifying potential new painkiller targets and the developing novel biological treatments for neuropathic pain.

### Nociceptor functionality

Anatomical and in vivo/in vitro electrophysiological data show that some nociceptors are myelinated Aδ-fibres and that others are unmyelinated C-fibres, different subsets of which are sensitive to a different range of stimuli, most being polymodal, but others responding to a narrower range of stimuli [2, 5]. Recent developments in transgenic mouse and imaging technology have led to elegant in vivo experiments using the genetically encoded Ca^2+^-indicator GCaMP [22–24], the fluorescence intensity of which is proportional to intracellular [Ca^2+^]. In contrast to electrophysiological studies, which suggest a predominantly polymodal nociceptor phenotype, some GCaMP studies have found that under control conditions, most sensory neurones in vivo are actually modality-specific, i.e., they respond to a single noxious stimulus, such as mechanical pinch of the hind paw, but not extreme heat or cold [22, 24]. However, like previous electrophysiology studies, in vivo GCaMP studies have found that injury and inflammation produce increased neuronal responsiveness [22–24]; the increased excitability of sensory neurones observed after injury is likely a key driver of both spontaneous and stimulus-evoked pain experienced by neuropathic pain patients. The discord between electrophysiological and GCaMP studies has been suggested to arise from the invasiveness of in vivo electrophysiological approaches and the trauma of dissociation required for in vitro analysis, both of which may induce inflammation contributing to the induction of nociceptor polymodality [22]. Although a technique that enables simultaneous measurement of multiple sensory neurones, in vivo measurement of intracellular [Ca^2+^] is, however, an indirect method of measuring action potential firing, i.e., signal conduction: not every action potential in a primary afferent fibre necessarily produces a measurable change in intracellular [Ca^2+^] at the cell body and not every change in intracellular [Ca^2+^] will necessarily result from action potential firing. Regardless of the limitations, in vivo Ca^2+^-imaging provides a powerful tool to enhance our understanding of how the nociceptive system works and the power of genetics means that it will be ever more possible to express a GCaMP indicator in a discrete neuronal subset to selectively determine its contribution to nociception and how this changes in neuropathic pain.

In addition to functional experiments, single-cell RNA-sequencing of sensory neurones has shown that neurones can be split into groups according to their transcriptome [25, 26], research that builds on earlier whole DRG RNA-sequencing studies that investigated how neuronal expression profiles change in neuropathic pain [27]. A significant advantage of single-cell RNA-sequencing is the unbiased nature of how data are produced. However, to harness the true power of single-cell RNA-sequencing, it should be conducted on sensory neurones of known anatomical innervation, i.e., through use of retrograde labelling techniques, because this technique has been used to show that neurones innervating different tissues have distinct properties. For example, NaV1.7 is expressed in NaV1.8-positive colonic neurones and yet mice lacking NaV1.7 in NaV1.8-positive neurones although having diminished somatic pain, experience normal visceral pain [28]; articular neurones have smaller acid responses, but greater likelihood of responding to ATP than cutaneous neurones [29], and of all 6 ASIC subunits, ASIC3 mediates acid-excitation of dural, corneal, and cardiac afferents, possibly contributing to the pain of headache, corneal pathologies, and cardiac ischaemia, respectively [30–32].

In the near future, it is likely that retrograde tracing will be coupled with RNA-sequencing to identify the molecular fingerprint of distinct neuronal subsets innervating different tissues. With such information to hand, different proteins and/or neuronal subsets can be targeted using transgenic mouse techniques to determine their contribution to neuropathic pain and thus identify novel analgesic targets.

### Unlocking nociceptor circuitry

Two further techniques recently utilised to further understanding of nociceptor function and circuitry are optogenetics and chemogenetics. Optogenetics involves inducing neuronal expression of light-activated membrane proteins whose activation can enable either depolarisation (e.g., channelrhodopsin-2, ChR2) or hyperpolarisation (e.g., halorhodopsin) to directly switch-on or switch-off neurones, respectively, as well as switching on of G protein-coupled receptor (GPCR) signalling cascades [33]. By contrast, chemogenetics entails, among other strategies, designer receptors exclusively activated by designer drugs (DREADDs), which are modified GPCRs no longer activated by their endogenous ligand acetylcholine, but instead the biologically inert substance clozapine-*N*-oxide (CNO, although recent work suggests that clozapine rather than CNO is the receptor ligand [34]); differently G protein-coupled DREADDs allow for activation (G_s_ or G_q_) or inhibition (G_i_) of neuronal function [35]. Using tissue-specific promoters, optogenetic and chemogenetic approaches can target distinct neuronal subsets. For example, plantar paw light stimulation in freely moving mice expressing ChR2 in NaV1.8-positive sensory neurones robustly produced nocifensive behaviours [36]. Like optogenetics, chemogenetics provides the potential to control neuronal function. For example, administration of CNO to mice expressing a G_i_-coupled DREADD in sensory neurones known to play a role in heat sensation increased the latency for paw withdrawal from a hot stimulus [37]. However, this study also raised concern about the use of DREADDs, because changes were observed in basal neuronal function, such as alterations in the amplitude of currents mediated by different NaV subunits. Therefore, results from DREADD studies hold great potential, but should be interpreted with caution and their future clinical use through gene therapy must control for certain risks. An alternative, but similar approach has recently been developed whereby viral transduction of an engineered chloride-channel sensitive to ivermectin (GluCl v2.0) was capable of long-term repeatable suppression of sensory neuron sensitivity and amelioration of neuropathic pain in rodents; the expression of GluCl v2.0 in human-induced pluripotent stem cell derived sensory neurones was also shown to cause their silencing in an ivermectin-dependent manner [38]. Overall, the ability to selectively control the excitability of neuronal subpopulations using either chemo- or optogenetic tools offers an attractive way of treating neuropathic pain compared to orally taken medications, which result in the whole body being exposed to the drug. Although such manipulations are likely still some way off, clinical trials for using ChR2 to treat advanced retinitis pigmentosa are underway [39].

Optogenetics and chemogenetics are also being used to further understanding about the circuitry of pain. In 1965, Melzack and Wall published the landmark paper *Pain Mechanisms: a new theory*, part of which suggested that input from low-threshold mechanoreceptors (LTMRs) inhibits nociceptor neurotransmission in the spinal cord [40]. Optogenetics has shown that ChR2 activation in a subset of myelinated A-fibre nociceptors required for mechanonociception produces an exaggerated pain response, which was mitigated when ChR2 was simultaneously expressed in LTMRs [41]. Co-activation of mechanonociceptors and LTMRs also resulted in fewer superficial dorsal horn neurones being activated compared to when mechanonociceptors were activated alone, which lends further support to Melzack and Wall’s theory that LTMR activation inhibits nociception. Studies such as this demonstrate the power of optogenetics for unravelling nociceptor neuronal circuitry, and in the next few years, optogenetics will likely be applied to determining how nociceptor circuitry becomes altered in neuropathic pain, which may identify potentially novel routes of analgesia, as well as, perhaps, providing explanations for side effects associated with current interventions.

## Clinical insights into neuropathic pain from pre-clinical research

As described in the Introduction, neuropathic pain is common and chronic pain sufferers often receive inadequate pain relief from current medications. A 2015 meta-analysis of trials looking at pharmacological treatments for neuropathic pain identified that although there is strong recommendation for the use of certain drugs (e.g., gabapentin, pregabalin, serotonin/noradrenaline reuptake inhibitors, and tricyclic antidepressants), the effects are relatively modest: the number needed to treat (NNT) to produce a 50% reduction in pain for those drugs with strong recommendation for use ranges from 3.6 to 7.7, i.e., 4–8 patients must be treated for one patient to experience at least 50% pain reduction when the placebo response is subtracted [42]. In addition to pharmacological treatment, a variety of other interventions are available for neuropathic pain, including: physical and psychological therapies, spinal cord stimulation, surgery, and transcranial magnetic stimulation (TMS), but here, discussion will be focused on the potential for the development of novel pharmacotherapy resulting from enhanced understanding of the molecular basis of neuropathic pain.

### Pre-clinical animal models

A major problem with regard to studying and treating neuropathic pain is its highly variable aetiology, ranging from brain lesions to demyelinating diseases of the spinal cord, and peripheral neuropathy resulting from conditions such as diabetes mellitus or as a result of anti-cancer chemotherapy. Pre-clinically, most studies are conducted on rodents and involve direct nerve injury, often to the sciatic nerve, which, although bearing poor resemblance to the case history of most neuropathic pain patients, does enable disease processes to be investigated and many treatments used clinically are able to reverse the pain behaviours observed in these models [43]. In addition to surgical models, there are also animal models that more closely resemble human disease pathogenesis, e.g., diabetic neuropathy [44] and certain chemotherapy treatments [45]. However, there has been a long-standing problem in efficiently translating analgesics from animal models through to the clinic, either due to lack of efficacy or due to side effects [46]. There are several potential reasons that have hindered translation of drugs, including: the highly varied aetiology of neuropathic pain in humans compared to the relatively restricted animal models, the in-bred (and thus genetically homogenous) animal subjects compared to the more genetically heterogeneous human population, and the difference in how pain assessed, i.e., most animal studies measure stimulus-evoked withdrawal behaviours, whereas neuropathic pain patients will often describe spontaneous pain. From the animal pain model perspective, it would, therefore, be advantageous to develop paradigms that measure changes in normal behaviour, e.g., suppression of burrowing can occur as a result of pain-induced changes in general well-being [47], alongside measurements of evoked pain. At the same time, it is, perhaps, necessary to try and better identify groups of patients for which a potential novel therapy may be of benefit when recruiting for clinical trials. Recent multicentre clustering analysis of quantitative sensory testing parameters measured in patients with different aetiologies has identified three distinct patient subgroups (sensory loss, thermal hyperalgesia and mechanical hyperalgesia), which may be underpinned by distinct pathological mechanisms. Thus, this may help to stratify patients for clinical trials testing analgesics arising from animal models where the mechanism of action is often well understood [48]. Subsequently, an algorithm has been developed to allocate patients to each subgroup, which should aid the stratification of patients both with regard to identifying appropriate patients for clinical trials and may enable easier identification of an individual patient’s optimal treatment regime [49].

Although animal models of neuropathic pain have their limitations and translation is currently far from perfect, such models have identified changes at all levels in the pain pathway in neuropathic pain, from alterations in sensory neurone protein expression to changes in spinal cord synaptic function and in the descending control of pain from the brain, some of which will now be discussed to shed light on potential future medications.

### Targeting action potential electrogenesis

From a therapeutic point of view, cutting off pain signals within the primary afferent nociceptor might be predicted to have fewer side effects than targeting a process within the central nervous system. To validate this approach, injection of the local anaesthetic lidocaine, which blocks all NaV subunits, at painful foci in patients suffering from painful neuropathy greatly diminished allodynia, suggesting that ongoing nociceptor input drives neuropathic pain [50]. What makes targeting of NaV subunits particularly appealing is that some neuropathic pain patients experience pain as a result of gain-of-function NaV mutations (for a review of these and other pain-related mutations, see [11–13]). The NaV1.7 subunit is commonly affected, mutations often causing a shift in the activation threshold (a smaller stimulus evokes pain) and/or slow inactivation (once activated the nerve fires for longer) [51, 52]. There are also NaV1.7 mutations that produce congenital insensitivity to pain [53, 54], but are not associated with serious systemic side effects (likely due to NaV1.7 expression being largely restricted to the peripheral nervous system), which suggests that selective inhibition of NaV1.7 could result in pain relief without producing a plethora of neurological side effects. Transgenic mouse models where NaV1.7 has been deleted in different neuronal subsets have demonstrated its critical contribution to different forms of pain [55], and recent work suggests that NaV1.7 activity also regulates endogenous opioid release, such that combining an NaV1.7 inhibitor with an opioid may provide synergistic analgesia with fewer side effects [56]. A recent Phase 2a trial in trigeminal neuralgia patients demonstrated that the selective NaV1.7 inhibitor BIIB074 produced fewer treatment failures than placebo and a better improvement in the daily pain score than placebo during the double-blind phase [57], results that are encouraging, but clearly larger trials are needed, especially those aimed at determining if BIIB074 is actually more efficacious and/or provides a lower side effect burden than the current first-line medications for trigeminal neuralgia, such as carbamazepine. Mutations in NaV1.8 can also underlie painful neuropathy in humans [58] and transgenic mouse work has identified a critical role for NaV1.8 in acute cold pain [59, 60], as well as cold allodynia in some neuropathic pain models [61]. Finally, mutations in NaV1.9 can cause both congenital insensitivity to pain [62] and painful neuropathy [63] in humans, and transgenic mouse work supports a role for NaV1.9 in inflammatory [64], neuropathic [61], and visceral pain [65], suggesting that like NaV1.7 and NaV1.8, NaV1.9 could be targeted to produce pain relief in certain neuropathic pain conditions. However, a key challenge for targeting any NaV subunit is to develop high enough subunit specificity to avoid off-target effects (e.g., inhibition of cardiac NaV1.5); a future possibility may be target modulators of NaV alpha subunits, rather than the alpha subunits themselves, for example beta subunits that modulate alpha subunit biophysical activity [66]. By contrast with inhibiting NaV subunits, there are numerous voltage-gated potassium channels (Kv) involved in sensory neurone action potential generation, which could also be targeted by a channel opener drug to relieve neuropathic pain [67, 68]. One promising example is retigabine, which is a Kv7 opener used as an anticonvulsant, but has also been shown to attenuate neuropathic pain in rodents [69]. However, a recent clinical trial looking at the effects of retigabine for treating postherpetic neuralgia, failed to find any difference compared to placebo in the primary endpoint of a change from baseline in the average pain score in the last 7 days of the maintenance phase [70].

In terms of action potential transmission, hyperpolarisation activated, cyclic nucleotide-gated ion channel (HCN) subunits are activated during the repolarization phase of action potential firing and are critical in returning a neurone to its resting membrane potential. The HCN2 subunit is predominantly expressed in sensory neurones and can be modulated by cyclic adenosine monophosphate (cAMP) to fire at more depolarised potentials, which induces trains of action potential firing [71]. The modulation of HCN2 by cAMP may well be important, because mice lacking HCN2 in a subset of sensory neurones fail to develop neuropathic pain [72] and ivabradine, which is a non-selective, peripherally restricted anti-anginal drug, reverses neuropathic pain in mice as effectively as gabapentin [73]. To date, however, no clinical trials have been published on the use of ivabradine for neuropathic pain. A potential complication lies in ivabradine’s non-selectivity, such that its blockade of cardiac HCN4 produces bradycardia at similar doses to those producing a relief from neuropathic pain [73], and thus, the search is on for a selective HCN2 inhibitor.

### Targeting transduction

As an alternative to reducing action potential firing through NaV or HCN blockade, the nociceptor transduction process itself could be disrupted. Mechanical hyperalgesia/allodynia is common features of neuropathic pain [48] and, in recent years, progress has been made in understanding how sensory neurones are activated by mechanical stimuli [74, 75]. The mechanically activated ion channel Piezo2 is necessary for normal touch sensitivity, but it is not involved in detecting noxious mechanical forces or in inflammatory mechanical hyperalgesia in mice [76]; it is, however, sensitised by signalling pathways associated with neuropathic pain [77]. Although Piezo ion channels are intrinsically mechanosensitive [78], the activation threshold for Piezo2 is reduced by stomatin-like protein 3 (STOML3) [79]. Studies in mice have shown that neuronal mechanosensitivity is lowered in neurones from mice lacking STOML3 [79, 80], that neuronal STOML3 expression is elevated in mouse neuropathic pain models [81] and mice lacking STOML3 show greatly reduced mechanical allodynia in neuropathic pain models [80, 81], i.e., targeting STOML3 may act to relieve the mechanical allodynia associated with neuropathic pain. The modulation of cellular mechanosensitivity by STOML3 is dependent upon its ability to form oligomers [79] and recent research identified two compounds that prevent oligomerisation and reverse neuropathic pain in mice [81], which highlights the tractability of such protein–protein interactions for pharmacotherapy. Compounds disrupting STOML3 oligomerisation prevented mechanical allodynia not only in nerve injury models, but also in a model of diabetic neuropathy [81], which raises hopes of possibly developing this therapeutic avenue for treating patients with diabetic neuropathy who experience a range of disturbances in mechanosensitivity, including dynamic mechanical allodynia [82].

### Interfering with central processing

In addition to targeting the primary afferent sensory neurone itself, an alternative is to target spinal cord neurotransmission between first- and second-order neurones. The CaV2.2 inhibitor ziconotide is used in the treatment of neuropathic pain and acts by blocking presynaptic CaV subunits to inhibit neurotransmission [83]. A major drawback is that to prevent systemic side effects, ziconotide must be administered intrathecally, which requires the implantation of a minipump. By determining the spinal cord pathology in neuropathic pain, it is hoped that more targeted therapies could be developed. Using rodent neuropathic pain models, it has been shown that the expression of glutamic acid decarboxylase (GAD, which synthesises the inhibitory neurotransmitter γ-aminobutyric acid, GABA) decreases following nerve injury [84]. Furthermore, some [85] but not all [86] studies have found that the number of GABAergic neurones decreases in neuropathic pain. With lowered GABAergic tone likely contributing to neuropathic pain it is thought that therapies which normalise GABAergic neurotransmission may be beneficial. Indeed, in rodents, positive modulation of α_2_ and/or α_3_ subunit containing GABA_A_ receptors reverses neuropathic pain [87]. However, a potential limitation of drugs that target GABA_A_ receptors is that they are widely expressed in the brain and thus may potentially produce unwanted side effects. To bypass this problem, recent work has shown that progenitors of GABAergic neurones can be transplanted into the spinal cord and integrate with the spinal cord circuitry to reverse nerve injury-induced and chemotherapy-induced neuropathic pain [88, 89]; unlike systemic injection of GABA receptor modulators that may modulate off-target GABAergic function, no migration of the progenitors outside of the segment into which injections were made was observed, thus diminishing the potential for side effects. Finally, over the last 15 years, research from several groups has also demonstrated a key interaction between spinal cord microglia and GABAergic function in neuropathic pain. Following the induction of neuropathic pain in rodents, spinal cord microglia are activated [90], and release brain-derived neurotrophic factor (BDNF) [91], which decreases neuronal K^+^/Cl^−^ co-transporter 2 (KCC2) expression [92]. Lowered KCC2 expression shifts the Cl^−^ concentration gradient, such that GABA_A_ receptor activation no longer causes inhibition, but can actually cause excitation of projection neurones [93]. An experimental compound has been identified that increases KCC2 surface expression, which normalises the intracellular Cl^−^ concentration, reverses mechanical allodynia in rats, and validates the prospect of targeting KCC2 to produce analgesia in neuropathic pain [94].

As well as diminished central inhibitory function in neuropathic pain, central circuits become hyperexcitable and understanding the mechanisms involved offers further potential for identifying new druggable targets for treating neuropathic pain [95]. One key process is long-term potentiation (LTP), an increase in synaptic strength following an elevated level of primary afferent input, which involves the glutamate *N*-methyl-D-aspartate (NMDA) receptor [96]. However, NMDA receptors are present throughout the nervous system and the non-analgesic effects of the recreational drug ketamine that blocks NMDA receptor activity (e.g., hallucinations and memory impairment), which demonstrate that systemic blockade of NMDA receptors is not a feasible route for standard neuropathic pain treatment, as such a peripherally restricted approach is likely to be necessary.

## Conclusions

In summary, neuropathic pain is a common condition with highly variably aetiology and for which the current treatments are often inefficacious and/or produce severe side effects. Pre-clinical research, largely using rodent models, but recently driven by human genetics, is focused on understanding how the nervous system changes during neuropathic pain in an effort to identify novel targets for analgesia and to validate their druggability. Rapid advances in technology have resulted in the development of many novel tools, such as chemo- and optogenetics, which will be applied to make further inroads into the pathogenesis of neuropathic pain using animal models, as well as having the potential to be developed clinically as highlighted by the current clinical trials with ChR2 in advanced retinitis pigmentosa. Finally, work in recent years has already identified several novel drug targets, such as NaV1.7, HCN2, STOML3, and KCC2, and future clinical trials will determine their true potential as targets for novel analgesics.
